# Social stress and risk of declining cognition: a longitudinal study of men and women in the United States

**DOI:** 10.1007/s00127-021-02089-7

**Published:** 2021-04-17

**Authors:** Jutta Lindert, Kimberley C. Paul, E. Lachman Margie, Beate Ritz, Teresa Seeman

**Affiliations:** 1 Department of Health and Social Work, University of Applied Sciences Emden/Leer, Constantiaplatz 4, 22687 Emden, Germany; 2 Women’s Research Center at Brandeis University, 415 South St., Waltham, MA 02453 USA; 3 Department of Epidemiology, Jonathan and Karin Fielding School of Public Health, University of California At Los Angeles, 650 Charles E. Young Dr. S, Los Angeles, CA 90095 USA; 4 The Heller School for Social Policy and Management, Brandeis University, 415 South St., Waltham, MA 02453 USA; 5 Division of Geriatrics, David Geffen School of Medicine, 10833 Le Conte Ave, Los Angeles, CA 90095 USA

**Keywords:** Social stress, Subjective social status, Discrimination, Episodic memory, Executive functioning

## Abstract

Limited research is available on the relationship between social stress and risk of declining cognition. We sought to examine whether social stress has adverse effects on risk of declining episodic memory and executive functioning in aging individuals. We used data from the MIDUS study, a national probability sample of non-institutionalized, English speaking respondents aged 25–74 living in the 48 contiguous states of the United States. The initial wave (1995) included 4963 non-institutionalized adults aged 32–84 (*M* = 55, SD = 12.4). We used an analytic sample from MIDUS-II (1996/1997) and MIDUS-III (2013) (*n* = 1821). The dependent variables are episodic memory and executive functioning, which were assessed with the Brief Test for Cognition (BTACT). The independent variables were social stress variables (subjective social status, family and marital stress, work stress and discrimination). To evaluate episodic memory and executive functioning changes over a time period of 10 years, we estimated adjusted linear regression models. Women report significantly lower subjective social status and more discrimination stress than men across all age groups. Controlling for education and income, age, and baseline episodic memory and executive functioning, lower subjective social status had additional adverse effects on declines in episodic memory in men and women. Marital risk had adverse effects on episodic memory in men but not in women. Daily discrimination had adverse effects on executive functioning on all individuals. Public health strategies should focus on reducing social stress in a socio-ecological perspective. Especially, subjective social status and discrimination stress might be a target for prevention efforts.

**Supplementary Information:**

The online version contains supplementary material available at 10.1007/s00127-021-02089-7.

## Introduction

Limited research is available on the relationship between social stress and risk of declining cognitive functioning in aging individuals. Declining cognitive functioning and dementia are major Public Health challenges [1]. Cognitive decline, however, varied widely. Furthermore, rates of cognitive decline are declining [2, 3]. The risk for a person to develop dementia over a lifetime is now 15% (95% confidence interval [CI] 7–9%) lower than it was in 2010 and incidence rates of dementia have declined over the past quarter century [2]. These findings of declining rates of dementia call for efforts to finding more causes for dementia and cognitive decline, although studies indicate that the age-specific incidence of cognitive decline is slowing down in the United States and other high-income countries [2, 4, 5]

Cognitive functioning includes cognitive abilities, such as episodic memory (EM) and executive functions (EFs). EM refers to the ability of learning, storing and recapturing about unique personal experiences over diverse periods ranging from minutes to years and decades [6]. EF includes a variety of abilities that enable goal‐directed behavior through strategy selection, information monitoring, and planning sequencing of actions [7]. EF includes stopping proponent or automatic responses, resisting distraction or interference from irrelevant information in the environment, switching between task sets, planning, monitoring, and verbal and design fluency. Performance in EM has been related to intact medial temporal lobe (MTL] and hippocampus structures [6]. By contrast, EF largely rely on the integrity of prefrontal and other frontal regions, and to some extent of parietal cortex [7]. 12 Modifiable factors for dementia and cognitive decline have been identified so far such as low education, hypertension, obesity, hearing loss, traumatic brain injury, alcohol abuse, smoking, depression, physical inactivity, social isolation, diabetes, and air pollution [8]. Despite the increasing knowledge on modifiable risk factors for cognitive decline, so far, we have limited knowledge about the long-term impact of social stress (subjective social status, social stress, and discrimination) on cognitive decline.

Subjective social status (SSS) or perception of rank on the social hierarchy is an important indicator of various health outcomes [9, 10] SSS is associated with a number of health outcomes [11, 12], including mortality [13], mental health [14], and cognition [15]. Indeed, the proposed physiological mechanisms underlying the relationship between SSS and health outcomes involve stress-related biological risk factors for disease, including altered cortisol response and reduced immune defense [16]. Many of these biological mechanisms are known risk factors for late-life cognitive impairment [17].

Social stress is conceptualized as perceived negative social exchanges such as conflict, rejection, criticism, and support failure [18, 19]. Accordingly, Social stress refers to the frequency and degree to which an individual experiences negative interactions with partner, family members, or friends. Social strain is associated with poor health outcomes (Lincoln 2000; Rook 1984; Sneed and Cohen 2014; Yang et al. 2014). Frequent negative social interactions with close others may lead to negative health outcomes, because negative interactions evoke stress responses, such as inflammation and sustained physiological activation, which may negatively affect health [20, 21].

Discrimination is a construct referring to behaviour resulting from the prejuidical attitudes, beliefs, and treatment of an individual or a group due to certain characteristics, such as gender social status, age, or race [22]. Discrimination can be defined as an act based on prejudices which results in “the differential treatment of individuals based on arbitrary or ascribed characteristics that are attributed to belonging to that group”. Discrimination stress has been associated with a variety of negative health outcomes [22–24]. Two meta‐analytic reviews indicate that discrimination stress is robustly associated with symptoms of depression and anxiety, coronary artery calcification, oxidative stress, shorter telomeres, dysregulations in cortisol, and inflammation [25–27], heightened physiologic and psychological stress responses that may have direct and indirect effects on cognitive function [28, 29], such as lower EM [30] and faster EM decline [16, 31]. Furthermore, studies suggest that discrimination is associated with greater oxidative stress [32], heightened physiological stress response [24], increased cortisol, dysregulation of the immune system and chronic non-specific inflammation. [16, 33], mortality risk [27, 34], and cardiovascular disease [35]. However, other studies do not show such an association [36].

Building upon previous cross-sectional studies which assessed social stress [18], and subjective social status [37], we assess the impact of social stress in an ecological model with stress on the individual level (subjective social status), on the partner and family level (social strain), on the work level (discrimination at work), and on the society level (discrimination). A better understanding of social factors contributing to cognitive decline can facilitate the development and evaluation of interventions to reduce inequalities in EM and EF decline and ultimately reduce the burden of cognitive decline in the aging population. The overall goal of the current study was, therefore, to expand understanding of the role of social stress in relation to declines in EM and EF. To accomplish this goal, we sought to [1] assess declines in EM and EF among men and women in the MIDUS cohort; [2] delineate variations of decline by level of social stress.

## Methods

### Sample

We use data from the MIDUS study. MIDUS is a national probability sample of non-institutionalized, English speaking respondents aged 25–74 living in the United States who were selected from households with a telephone; enrolment began in 1995 (MIDUS I). The initial wave included 4963 non-institutionalized adults aged 32–84 (*M* = 55, SD = 12.4) from the 48 contiguous states. The sample was obtained using random digit dialing with a response rate of 71%. Retention rates in subsequent waves were slightly higher among women, whites, married people, and people with more education and better health (Radler and Ryff 2010). Measures of cognition were collected in MIDUS II and III only, thus, here we use data on SSS and discrimination from MIDUS II and cognition data from MIDUS II and III. MIDUS was approved by the Institutional Review Boards of the participating institutions, and informed consent was obtained. Our analytical sample consists of *N* = 1821 individuals.

### Measures

The dependent variables are episodic memory [EM) and executive functioning (EF). The primary independent variables of interest are social stress assessed as SSS, social strain in family and work and discrimination.

Cognitive function in MIDUS II and III was assessed using the Brief Test of Adult Cognition by Telephone (BTACT). [38] The BTACT was designed especially to enable assessment of cognitive functioning in large community-based samples [39] and to identify non-pathological variation in cognitive function. Participants were asked to complete a series of cognitive tests after a brief hearing test. The BTACT includes EM and EF. EM is assessed by immediate and delayed recall trials from the Rey Auditory—Verbal Learning Test [40]. EF is assess by the Category—Verbal Fluency Test [41], the Digit Span Backward Test [42], the Number Series [43], the 30 s and Counting Tasks [39] and the Stop and Go Switch Task [39]. Composite scores for EM and EF were computed as mean *z*-scores based on the means and SDs at M2.

The independent variables are validated scales of social strain [44, 45] Social strain at the family level was measured using four indices of negative social interaction with spouse/partner, friends, and family members. All items were answered on a 4-point scale ranging from one (often) to four (never). Items included, “How often do they criticize you?”, “How often do they make too many demands on you?”, “How often do they let you down when you are counting on them?”, “How often do they get on your nerves?” Two additional items were included to assess social strain from partner/spouse: “How often does he or she (i.e., partner/spouse) argue with you?” and “How often does he or she (i.e., partner/spouse) make you feel tense?” Measures of social strain at the work level include perceived inequality at work (6 items) and chronic job discrimination (6 items). SSS includes perceived inequality (6 items). Discrimination was evaluated as lifetime discrimination (11 items) and daily discrimination (9 items). (Supplemental Material Part A. Scales). Items included, “You are treated with less courtesy or respect than other people,” “You receive poorer service than other people at restaurants or stores,” “People act as if they think you are not smart,” “People act as if they are afraid of you,” and “You are threatened or harassed.” In the current study, mean scores on the scale were reversed prior to analysis so that higher scores correspond to greater everyday discrimination.

Age (in years) corresponded to age at the time of MIDUS II and III. Gender was quantified as dichotomous (male/female). Self-reported race/ethnicity was dummy-coded into four categories: non-Hispanic White, non-Hispanic Black, Hispanic (of any race), and non-Hispanic other. The largest category, non-Hispanic White, was treated as the reference group. We also control for the following variables: baseline cognition *z*-score, relationship status (living vs. not living with partner), educational attainment (some college or more vs. high school or less), income level at baseline (per 100% of federal poverty level (FPL), accounting for household size), employment status (unemployed or retired, vs. employed), perceived physical health (yes/no scale), physical activity (vigorous vs. none), and depression and anxiety scores (continuous scales).

### Statistical methods

We estimated cognition means and calculated differences across four age categories (40–49, 50–59, 60–69, ≥ 70) using cognition *z*-scores (EM, EF). The discrimination- and social stress and strain scales were compared using the Wilcoxon–Mann–Whitney Rank Sum test and trends were evaluated using the Cochrane–Armitage test. We compared changes in scores from MIDUS II and III using paired *t* tests and we calculated Pearson correlation coefficients. We modeled cognition (EM and EF *z*-scores) cross-sectional with a linear regression model. Since the inequality scales were ordinal in nature (1 often; 2 sometimes; 3 rarely; 4 never) and ‘never’ was a rare response, we generated a three-level variable (1 often; 2 sometimes; 3 rarely or never). For discrimination we used a continuous scale. Regression models were adjusted throughout for baseline age, race/ethnicity, education, living with partner, income and employment status, physical activity, depression and anxiety. We included all age groups as analyses revealed no clear differences of results by baseline age group, that is, no differences between cohorts who were in midlife or late life at baseline.

To model change in cognition between MIDUS II and III, we created a variable of average change in cognition over 10 years (MIDUS II–MIDUS III)/years of follow-up) × 10). Both EM and EF change scores were created and modeled with linear regression adjusting for all factors listed above plus the baseline (MIDUS II) cognition score. In sensitivity analysis, we also restricted to Caucasians only and stratified according to income (higher (> median) vs. lower (≤ median). We controlled for self-rated health (data now shown). However, there was no statistically significant effect.

To statistically assess the interaction between gender and discrimination, we including a gender × discrimination interaction term in our linear regression models.

## Results

Means and standard deviations of social stress variables are presented in Table 1. Women report more family strain than men do across all age groups. Participants report less marital strain with increasing age. Spouse/partner strain is reported more often by women than men, while work stress is reported more often by men. Work inequality is inversely associated with increasing age for men but not for women. Additionally, discrimination stress is reported less often with increasing age. Finally, lifetime- and daily discrimination stress is higher in women than men across all age groups. Men reported more chronic job discrimination than women.

**Table 1:** Stress/strain variables by sex and age at MIDUS 2

Stress/Strain variables	Age range MIDUS 2	Men			Women			*P* value
		*N*	Mean (SD)	*P* for age trend	*N*	Mean (SD)	*P* for age trend	
Marital strain	< 40	64	3.3 (1.6)	< .0001	82	3.1 (1.6)	< 0.0001	0.39
	40–49	210	3.3 (1.6)		265	3.3 (1.7)		0.73
	50–59	286	3.0 (1.4)		293	3.2 (1.7)		0.52
	60–69	209	2.7 (1.1)		216	2.8 (1.3)		0.60
	≥ 70	100	2.4 (0.9)		96	2.6 (1.2)		0.49
Family strain	< 40	74	2.1 (0.5)	< .0001	109	2.3 (0.5)	< 0.0001	0.008
	40–49	253	2.0 (0.6)		325	2.2 (0.6)		0.001
	50–59	336	2.0 (0.5)		389	2.1 (0.6)		0.004
	60–69	234	1.9 (0.5)		321	2.0 (0.5)		0.005
	≥ 70	119	1.8 (0.4)		166	1.9 (0.5)		0.14
Spouse/partner strain	< 40	64	2.1 (0.6)	0.0003	82	2.1 (0.6)	0.8627	0.80
	40–49	210	2.2 (0.6)		265	2.2 (0.7)		0.70
	50–59	286	2.1 (0.5)		294	2.2 (0.7)		0.06
	60–69	209	2.0 (0.5)		215	2.1 (0.7)		0.08
	≥ 70	100	2.0 (0.5)		95	2.2 (0.6)		0.23
Work level stress								
Perceived Inequality at work	< 40	72	1.7 (0.6)	< .0001	88	1.6 (0.5)	0.0224	0.17
	40–49	234	1.6 (0.5)		272	1.6 (0.5)		0.69
	50–59	304	1.5 (0.5)		297	1.5 (0.5)		0.85
	60–69	161	1.4 (0.5)		168	1.5 (0.5)		0.004
	≥ 70	40	1.4 (0.5)		34	1.5 (0.5)		0.46
Chronic job discrimination	< 40	72	12.3 (4.8)	< .0001	88	10.7 (4.5)	< 0.0001	0.02
	40–49	233	11.7 (4.5)		271	10.7 (4.2)		0.01
	50–59	305	10.8 (4.2)		297	10.2 (4.3)		0.04
	60–69	162	8.9 (3.6)		170	9.0 (3.5)		0.65
	≥ 70	40	8.4 (3.8)		37	8.3 (3.8)		0.90
Society level stress								
Perceived Inequality of family	< 40	52	1.4 (0.5)		78	1.6 (0.6)		0.04
	40–49	192	1.6 (0.5)		267	1.5 (0.5)		0.37
	50–59	294	1.5 (0.5)		343	1.6 (0.5)		0.0002
	60–69	208	1.5 (0.4)		305	1.6 (0.5)		0.05
	≥ 70	110	1.5 (0.5)	0.3103	158	1.6 (0.5)	0.1149	0.41
Lifetime discrimination	< 40	74	1.0 (1.7)	0.002	109	1.0 (1.5)	0.0003	0.60
	40–49	253	0.9 (1.5)		327	1.0 (1.4)		0.03
	50–59	337	0.8 (1.3)		389	1.1 (1.6)		0.01
	60–69	237	0.7 (1.3)		323	0.9 (1.4)		0.22
	≥ 70	120	0.4 (1.1)		167	0.5 (1.0)		0.27
Daily discrimination	< 40	73	13.7 (5.6)	0.0002	109	13.4 (4.6)	< 0.0001	0.99
	40–49	251	13.1 (4.7)		326	13.8 (4.5)		0.03
	50–59	336	12.4 (4.7)		387	13.0 (4.2)		0.004
	60–69	236	12.0 (4.1)		316	12.0 (3.7)		0.39
	≥ 70	118	11.9 (3.6)		164	11.3 (3.3)		0.12

All items coded so that high scores reflect higher standing in each scale

*P* value based on Wilcoxon–Mann–Whitney (Rank Sums), comparing men to women

P for trend comparing for trend across age within sex, i.e.,, does age group predict stress/strain variable (age cat treated as linear)

Women show less decline in EM compared with men, but, men show less decline in EF, and both EM and EF decrease with increasing age (Lachman et al. 2014; Hughes et al. 2018). (Supplemental Material Table 1) Decrease in EM in higher income women is slower than in lower income men and women experience faster EF decreases; in fact, EF decrease is fastest in higher income women age 70 + compared with all other groups (Supplemental Material Table 2).

Table 2 presents information on socio-demographics, and health characteristics [*N* (percentage) or mean (SD)], and how they predict changes in EM and EF. Age, EM, and EF at baseline predict EM and EF changes.

**Table 2:** Stress/strain variables and predicting change in episodic memory change

Scales	Tertile	Episodic memory							
		Model 1: Men (*n* = 785–984)		Model 2: Men, Excluding unemployed/retired		Model 1: Women (*n* = 818–1234)		Model 2: Women, Excluding unemployed/retired	
		*β* (SE)	*P* value	*β* (SE)	*P* value	*β* (SE)	*P* value	*β* (SE)	*P* value
Family and Marital level stress									
Perceived Inequality of family	0	Ref		Ref		Ref		Ref	
	1	0.06 (0.06)	0.33	0.08 (0.07)	0.26	0.05 (0.07)	0.45	0.01 (0.09)	0.89
	2	0.20 (0.10)	0.04	0.32 (0.12)	0.008	0.29 (0.09)	0.001	0.30 (0.12)	0.01
Marital strain	0	Ref		Ref		Ref		Ref	
	1	– 0.14 (0.07)	0.05	– 0.16 (0.08)	0.05	– 0.07 (0.09)	0.46	– 0.21 (0.11)	0.05
	2	0.13 (0.06)	0.03	0.18 (0.07)	0.01	0.04 (0.07)	0.58	0.01 (0.08)	0.89
Family strain	0	Ref		Ref		Ref		Ref	
	1	0.07 (0.10)	0.50	0.05 (0.12)	0.66	0.08 (0.12)	0.49	0.23 (0.17)	0.18
	2	0.08 (0.10)	0.42	0.06 (0.12)	0.62	0.12 (0.12)	0.33	0.25 (0.17)	0.15
Spouse/partner strain	0	Ref		Ref		Ref		Ref	
	1	0.18 (0.22)	0.41	0.29 (0.27)	0.28	– 0.13 (0.16)	0.42	– 0.06 (0.19)	0.73
	2	0.23 (0.22)	0.30	0.31 (0.27)	0.26	– 0.07 (0.16)	0.65	– 0.14 (0.18)	0.45
Work level stress									
Perceived Inequality at work	0	Ref		Ref		Ref		Ref	
	1	0.11 (0.06)	0.10	0.08 (0.07)	0.25	– 0.07 (0.08)	0.40	– 0.07 (0.08)	0.39
	2	0.13 (0.09)	0.13	0.13 (0.10)	0.19	0.06 (0.11)	0.57	0.07 (0.12)	0.58
Chronic job discrimination	0	Ref		Ref		Ref		Ref	
	1	– 0.05 (0.07)	0.43	– 0.10 (0.07)	0.19	0.02 (0.08)	0.83	0.03 (0.08)	0.68
	2	– 0.06 (0.07)	0.34	– 0.09 (0.07)	0.22	– 0.02 (0.08)	0.78	0.01 (0.09)	0.96
Society level stress									
Perceived inequality of family	0	Ref		Ref		Ref		Ref	
	1	0.06 (0.06)	0.33	0.08 (0.07)	0.26	0.05 (0.07)	0.45	0.01 (0.09)	0.89
	2	0.20 (0.10)	0.04	0.32 (0.12)	0.008	0.29 (0.09)	0.001	0.30 (0.12)	0.01
Lifetime discrimination	cont'	0.02 (0.02)	0.31	0.01 (0.02)	0.81	– 0.03 (0.02)	0.17	– 0.01 (0.02)	0.51
Daily discrimination	cont'	0.01 (0.01)	0.10	0.03 (0.01)	0.51	0.01 (0.01)	0.21	0.02 (0.01)	0.73

Outcome: (MIDUS2 Cognition—MIDUS3 Cognition)/follow-up time)*10. Higher score represents more decline, negative score means less decline from baseline cognition score

*All models control for age at MIDUS 2, baseline cognition score, race/ethnicity (White, Hispanic, other), education (some college or more vs. high school of less), living with partner, income (per 100% above FPL), unemployed (vs. employed), retired (vs. employed), physical health (self-reported), vigorous physical activity, depression (continuous scale), anxiety (continuous scale)

Table 3 shows that stress scores predict changes in EM. More marital stress is associated with decreased EF in men. More perceived work inequality stress is cross-sectional associated with worse EM in men but not in women. At the societal level, more daily discrimination stress is associated with worse EM and more lifetime and daily discrimination with worse EF in men.

**Table 3:** Stress/strain variables and predicting change in executive function change

Scales	Tertile	Executive function							
		Model 1: Men (*n* = 785–984)		Model 2: Men, excluding unemployed/retired		Model 1: Women (*n* = 818–1234)		Model 2: Women, excluding unemployed/retired	
		*β* (SE)	*P* value	*β* (SE)	*P* value	*β* (SE)	*P* value	*β* (SE)	*P* value
Family and Marital level stress									
Marital strain	0	Ref		Ref		Ref		Ref	
	1	– 0.03 (0.05)	0.45	– 0.03 (0.05)	0.55	0.01 (0.05)	0.91	– 0.02 (0.05)	0.69
	2	0.01 (0.04)	0.82	0.01 (0.04)	0.86	0.05 (0.03)	0.16	0.09 (0.04)	0.03
Family strain	0	Ref		Ref		Ref		Ref	
	1	– 0.04 (0.06)	0.57	– 0.01 (0.07)	0.86	– 0.04 (0.07)	0.52	– 0.05 (0.08)	0.56
	2	0.02 (0.07)	0.73	0.03 (0.07)	0.70	0.002 (0.07)	0.97	0.002 (0.09)	0.98
Spouse/partner strain	0	Ref		Ref		Ref		Ref	
	1	– 0.04 (0.14)	0.79	– 0.09 (0.15)	0.57	– 0.01 (0.08)	0.86	– 0.05 (0.09)	0.62
	2	– 0.05 (0.14)	0.71	– 0.09 (0.15)	0.58	– 0.003 (0.08)	0.97	– 0.01 (0.09)	0.88
Work level stress									
Perceived Inequality at work	0	Ref		Ref		Ref		Ref	
	1	0.03 (0.04)	0.44	0.02 (0.04)	0.69	– 0.00 (0.04)	0.99	0.004 (0.04)	0.92
	2	0.05 (0.05)	0.33	0.03 (0.06)	0.64	0.04 (0.06)	0.50	0.04 (0.06)	0.49
Chronic job discrimination	0	Ref		Ref		Ref		Ref	
	1	– 0.01 (0.04)	0.87	0.01 (0.04)	0.80	– 0.03 (0.04)	0.46	– 0.01 (0.04)	0.72
	2	– 0.01 (0.04)	0.77	0.01 (0.04)	0.85	0.04 (0.04)	0.36	0.05 (0.04)	0.28
Society level stress									
Perceived Inequality of family	0	Ref		Ref		Ref		Ref	
	1	– 0.02 (0.04)	0.58	– 0.05 (0.04)	0.22	0.04 (0.04)	0.31	0.06 (0.04)	0.14
	2	0.09 (0.06)	0.17	0.04 (0.07)	0.58	0.08 (0.05)	0.11	0.06 (0.06)	0.31
Lifetime discrimination	cont'	0.004 (0.01)	0.77	0.01 (0.01)	0.37	– 0.001 (0.01)	0.94	0.01 (0.01)	0.53
Daily discrimination	cont'	0.01 (0.004)	0.001	0.01 (0.004)	0.005	0.004 (0.004)	0.25	0.004 (0.004)	0.35

Outcome: (MIDUS2 Cognition—MIDUS3 Cognition)/follow– up time) × 10. Higher score represents more decline, negative score means less decline from baseline cognition score

*All models control for age at MIDUS 2, baseline cognition score, race/ethnicity (White, Hispanic, other), education (some college or more vs. high school of less), living with partner, income (per 100% above FPL), unemployed (vs. employed), retired (vs. employed), physical health (self-reported), vigorous physical activity, depression (continuous scale), anxiety (continuous scale)

Social stress is associated with EM in women and men (Table 2). However, the specific types of stress are associated differently with EM. Marital stress is associated with EM decline in men, but not in women. Additionally, daily discrimination stress is associated with worse EF in men but not in women. In working women, marital stress is associated with declines in EF. However, low SSS is inversely associated with EM and EF in high-income men and low-income women.

Lower SSS is associated faster declines in EM adjusted for age, education, income and baseline cognition score in all individuals in our sample (Table 3). Less decline in EF is inversely associated with daily discrimination. More decline in EF is associated with daily discrimination in low income men. Adults who were exposed to higher levels of daily discrimination showed significantly more decline in (Supplemental Material Table 4). Work stress was not related to more decline in EM and EF.

**Table 4:** Discrimination variables and cognitive function change, showing the interaction between men and women

Model Term		Executive Function		Episodic Memory	
		*β* (SE)	*P* value	*β* (SE)	*P* value
Outcome: (MIDUS2 Cognition—MIDUS3 Cognition)/follow-up time)*10. Higher score represents more decline, negative score means less decline from baseline cognition score					
Gender reference is women, meaning beta refers to how men are different from women					
Discrimination (continuous measures)					
Lifetime discrimination model	Lifetime discrimination	– 0.001 (0.01)	0.91	– 0.02 (0.02)	0.21
	Male	– 0.01 (0.03)	0.77	0.33 (0.05)	< .0001
	Discrimination*Male	0.01 (0.01)	0.52	0.03 (0.03)	0.24
Daily discrimination model	Daily discrimination	0.003 (0.003)	0.46	0.01 (0.01)	0.08
	Male	– 0.14 (0.06)	0.03	0.39 (0.11)	0.0004
	Discrimination*Male	0.01 (0.005)	0.03	– 0.004 (0.008)	0.66

### Sensitivity analyses

We conducted sensitivity analyses to investigate whether results are stable across population subgroups (Caucasian only vs. other, higher income vs. lower income) (Supplemental Material Table 5). The sensitivity analyses suggest effects of marital and spouse stress are higher for higher income men compared to lower income men. Additionally, SSS is associated with worsening EM for higher income men and lower income women. The sensitivity analyses suggest, furthermore, that daily discrimination stress has an effect on EF lower income men—especially on Caucasians (Supplemental Material Table 6).

## Discussion

The goal of the current study was to expand understanding of the role that social stress may play declines in EM and EF. This longitudinal study provides evidence that among social stress factors SSS and discrimination are associated with declines in in EM and EF, independent of known risk factors for cognitive decline [8, 46]. Together, these results suggest that SSS and discrimination may be additional modifiable factors to prevent declines in EM and EF.

Our data suggest that SSS, social strain and discrimination are associated with declines in EM and EF. Our results showed that those with lower education had higher odds of declining EM. This finding is in line with recent findings on the effects of education on less decline in cognition [8, 46]. These studies support the theory of cognitive reserve [47] which suggest that educational attainment may supply a set of factors that reduces the age-related changes in the brain and increase brain plasticity [48]. Additionally, our data suggest that independent of the known factor of education social stress has an impact on declines in EM and EF. These findings are consistent with experimental evidence suggesting that the psychological repercussions of perceived inequality have adverse effect on health. Indeed, research indicates that, independent of objective SES, SSS predicts mental and physical health outcomes. SSS has been described as a more comprehensive measure of one’s social position than education or income, because it reflects an individual’s “cognitive averaging” of multiple dimensions of SES as well as other status-related information, such as perceived control, feelings of financial security, and discrimination [10, 49]. The mechanisms why SSS may have an impact on EM and EF might be explained through experimental evidence. Studies suggest that low SSS elevates multiple biological risk factors for disease. Interestingly, in our study higher income men and lower income women drive these associations between SSS, EM, and EF.

We found that discrimination is a further factor that has a negative effect on EM and EF. These findings are consistent with the cumulative disadvantage theory, which proposes that adults’ exposure to lower SSS and discrimination results in wear and tear effects over time [50, 51]. According to this model, adults are affected by social stress during lifetime. Research suggests that social stress can contribute to chronic inflammation, and increased risk of psychiatric disorders [19, 52, 53]. Our findings contribute to the literature by suggesting that social stress not only contributes to health but to changes (declines) in EM and EF.

Our study also suggests that the effects of social stress are different for men and women in the United States. SSS has an impact on men and women, but specifically on high-income men and low-income women. High-income men and low-income women may perceive SSS trying to offer opportunities for their families. In men, the traditionally gendered roles of provider of opportunities, is a fundamental aspect of men’s identities [54]. Despite the changes and flexibility in gender roles over time, the family provider role continues to be an important aspect of men’s identity [55]. Likewise, for low—income women, which are mostly the main earners of family income—the role of provider of opportunities similarly applies and it`s stress affects EM and EF in these women.

Socioeconomic inequalities expressed in objective measures of income and education have a well-known influence on cognitive outcomes [56]. Our study add knowledge that social stress, specifically SSS and discrimination are additionally contributing to declines in EM and EF Our finding is in line with the Religious Orders Study and Rush Memory and Aging Project which suggests that social stress doubles the risk for cognitive decline in old age [57, 58]. The potential link with chronic stress and premature aging is also reported in the context of biological changes such as telomere shortening, increased inflammatory responses, microglial activation and increased oxidative stress [59]. There are several methodological challenges in investigating risk factors for cognitive decline such as cardiovascular factors and social stress. Stress may come and go; however, negative consequences of social stress may be pervasive. Lower levels of social stress has been shown to benefit memory and executive performance and negative interactions may increase stress and have a negative impact on overall cognitive function including episodic memory [60].

Although this study was able to take advantage of the longitudinal and multidimensional nature of MIDUS data, it was limited in some respects. There is, like in many longitudinal studies, evidence of attrition bias in MIDUS. With retention rates in subsequent waves being higher among women, whites, married people, and people with more education and better health. Specifically, as compared to those who remained, participants no longer in the MIDUS III had worse self-rated health, greater socioeconomic disadvantage, were less likely to have current employment, reported greater discrimination, were more likely male, individual of a racial/ethnic minority, not married, and were more likely to report greater negative affect and more neuroticism at the T1 assessment. This does not affect the internal validity of our study; however, the effects of discrimination on cognition may even be more severe as the population of our study experiences potentially less discrimination than the general US population. Additionally, we cannot exclude reporting bias. Respondents may report social stress when none actually occurred; Second, MIDUS does not ask respondents about exposure to social stress before the first measurement (*t*_0_), so we were unable to examine any type of social stress prior to their baseline interview. Furthermore, even though we are able to examine experiences across family, work and society, the domains explored do not represent the full range of places and circumstances, where social stress and discrimination and perceived inequality can be experienced. Despite these measurement challenges requiring more methodological research, self-reported social stress and discrimination research remains an important domain of inquiry. Given these measurement limitations, results presented here may underestimate the effects of social stress on declines in EM and EF. Additionally, MIDUS is fully population based at baseline but as in almost all longitudinal studies less healthy individuals dropped out over the years slightly more. Therefore, the results should be replicated in studies, including in further countries. It might be that the findings are not fully generalizable to individuals living in other countries. Finally, this study used a brief, telephone-based cognitive assessment, which might be expanded in further studies.

Limitations notwithstanding, this study makes new contributions to the literature. The current finding that social stress was associated with declines in EM indicates that more modifiable risk factors for declines in cognition might play a critical role for cognitive health. These social risk factors may represent novel targets for the prevention of cognitive morbidity among older adults. The findings of this study extend our understanding risk factors for cognition and highlight the prominence of social stress in shaping cognition.

## Supplementary Information

Below is the link to the electronic supplementary material.

Supplementary file1 (DOCX 131 kb)
