# Supplementary material for: Social stress and risk of declining cognition: a longitudinal study of men and women in the United States
Source: Soc Psychiatry Psychiatr Epidemiol. 2021 Apr 17;57(9):1875–84. doi: 10.1007/s00127-021-02089-7 (PMC8522181; doi:10.1007/s00127-021-02089-7)
Supplement: Supplementary file 1 — Supplementary file1 (DOCX 131 kb) [file 127_2021_2089_MOESM1_ESM.docx]

**Supplemental Material Part A and Part B**

**Supplemental Material. Part A. Scales**

**MARITAL STRAIN**

**Marital risk scale [B1SMARRS]:**

Items: 2 items - Self-Administered Questionnaire, Section L, Question 7, 8.

7. “During the past year, how often have you thought your relationship might be in trouble?”

Coding: 1 Never; 2 Once; 3 A few times; 4 Most of the time; 5 All the time.

8. “(Realistically) what do you think the chances are that you and your partner will eventually separate?” (R)

Coding: 1 Very likely; 2 Somewhat likely; 3 Not very likely; 4 Not likely at all.

Scaling: The scale is constructed by calculating the sum of the values of the two items. Items marked with (R) were reverse-coded so that higher values indicate higher levels of marital risk.

Source(s): Booth, A., Johnson, D., & Edwards, J. N. (1983). Measuring marital instability. Journal of Marriage and the Family, 45, 2, 387-394.

Category Construction: Low (1-2), Mid (3), High (4-9)

**FAMILY STRAIN**

**Family Strain [B1SKINNE]:**

Items: 4 items - Self-Administered Questionnaire, Section J, Questions 2 (g – j).

g. “Not including your spouse or partner, how often do members of your family make too many demands on you?”

h. “How often do they criticize you?”

i. “How often do they let you down when you are counting on them?”

j. “How often do they get on your nerves?”

Coding: 1 Often; 2 Sometimes; 3 Rarely; 4 Never.

Scaling: Scales are constructed by calculating the mean of the values of the items in each scale. Items were reverse-coded so that high scores reflect higher standing in the scale.

Source(s): Schuster, T. L., Kessler, R. C., & Aseltine, R. H. (1990). Supportive interactions, negative interactions, and depressive mood. American Journal of Community Psychology, 18: 423-438.: MIDUS scales are revised from this study.

Whalen, H. R., & Lachman, M. E. (2000). Social support and strain from partner, family and friends: Costs and benefits for men and women in adulthood. Journal of Social and Personal Relationships, 17, 1, 5-30.

Category Construction: Low (≤1), Mid (1-2), High (>2)

**SPOUSE / PARTNER SUPPORT AND STRAIN**

**Spouse/Partner Strain [B1SSPCRI]:**

Items: 6 items -Self-Administered Questionnaire, Section L, Questions 11 (g – l).

g. “How often does your spouse or partner make too many demands on you?”

h. “How often does he or she argue with you?”

i. “How often does he or she make you feel tense?”

j. “How often does he or she criticize you?”

k. “How often does he or she let you down when you are counting on him or her?”

l. “How often does he or she get on your nerves?”

Coding: 1 Often; 2 Sometimes; 3 Rarely; 4 Never.

Scaling: Scales are constructed by calculating the mean of the values of the items in each scale. Items were reverse-coded so that high scores reflect higher standing in the scale.

Source(s): Grzywacz, J. G., & Marks, N. F. (1999). Family solidarity and health behaviors: Evidence from the National Survey of Midlife Development in the United States. Journal of Family Issues, 20, 2, 243-268.

Schuster, T. L., Kessler, R. C., & Aseltine, R. H. (1990). Supportive interactions, negative interactions, and depressive mood. American Journal of Community Psychology, 18: 423-438.: MIDUS scales are revised from this study.

Whalen, H. R., & Lachman, M. E. (2000). Social support and strain from partner, family and friends: Costs and benefits for men and women in adulthood. Journal of Social and Personal Relationships, 17, 1, 5-30.

Category Construction: Low (≤1), Mid (1-2), High (>2)

**PERCEIVED INEQUALITY IN WORK**

**Perceived inequality in work [B1SPIWOR]:**

Items: 6 items - Self-Administered Questionnaires, Section F; Questions 32 (a – f).

a. “I feel cheated about the chances I have had to work at good jobs.” (R)

b. “When I think about the work I do on my job, I feel a good deal of pride.”

c. “I feel that others respect the work I do on my job.”

d. “Most people have more rewarding jobs than I do.” (R)

e. “When it comes to my work life, I’ve had opportunities that are as good as most people’s.”

f. “It makes me discouraged that other people have much better jobs that I do.” (R)

Coding: 1 A lot; 2 Some; 3 A little; 4 Not at all

Scaling: The scale is constructed by calculating the mean of six items. Items marked with (R) were reverse-coded so that high scores reflect higher standing in the scale.

Source(s): Corey L. M. Keyes. Studies using the scales: Ryff, C. D., Magee, W. J., Kling, K. C., & Wing, E. H. (1999). Forging macro-micro linkages in the study of psychological well-being. In C.D. Ryff & V.W. Marshall (Eds.), The self and society in aging processes (pp.247-278). New York: Springer Publishing.

Category Construction: Low (≤1), Mid (1-2), High (>2)

**CHRONIC JOB DISCRIMINATION [B1SJOBDI]:**

Items: 6 items - Self-Administered Questionnaires, Section F; Questions 31 (a – f)

a. “How often do you think you are unfairly given the jobs that no one else wanted to do?”

b. “How often are you watched more closely than other workers?”

c. “How often does your supervisor or boss use ethnic, racial, or sexual slurs or jokes?”

d. “How often do your coworkers use ethnic, racial, or sexual slurs or jokes?”

e. “How often do you feel that you are ignored or not taken seriously by your boss?”

f. “How often has a co-worker with less experience and qualifications gotten promoted before you?”

Coding: 1 Once a week or more; 2 A few times a month; 3 A few times a year; 4 Less than once a year; 5 Never.

Scaling: The scale is constructed by calculating the sum of the values of the items. Items were reverse-coded so that high scores reflect higher standing in the scale. For an item with a missing value, the mean value of completed items is imputed.

Source(s): D. Williams.

Category Construction: Frequently (≤8), Infrequently (>8)

**PERCEIVED INEQUALITY IN FAMILY**

**Perceived Inequality in Family (Relationship with Children) [B1SPIFAM]:**

Items: 6 items - Self-Administered Questionnaire, Section K, Question 6 (a-f).

a. “I feel good about the opportunities I have been able to provide for my children.” (R)

b. “It seems to me that family life with my children has been more negative than most people's.”

c. “Problems with my children have caused me shame and embarrassment at times.”

d. “As a family, we have not had the resources to do many fun things together with the children.”

e. “I believe I have been able to do as much for my children as most other people.” (R)

f. “I feel a lot of pride about what I have been able to do for my children.” (R)

Coding: 1 Not at all true; 2 A little true; 3 Moderately true; 4 extremely true.

Scaling: The scale is constructed by calculating the mean of the values of the items. Items marked with (R) were reverse-coded so that high scores reflect higher standing in the scale.

Source(s): Ryff, C. D., Magee, W. J., Kling, K. C., & Wing, E. H. (1999). Forging macro-micro linkages in the study of psychological well-being. In C.D. Ryff & V.W. Marshall (Eds.), The self and society in aging processes (pp.247-278). New York: Springer Publishing.

Studies using the scales: Lachman, M. E., & Weaver, S. L. (1998). Sociodemographic variations in the sense of control by domain: Findings from the MacArthur Studies of Midlife. Psychology and Aging, 13, 4, 553-562.

Notes:

- The study of perceived inequalities follows from the observation that individuals live in social worlds that are filled with conspicuous symbols of class standing (e.g., occupation, car, clothing, home, leisure activities). MIDUS perceived inequality questions were designed to assess the extent to which individuals have an awareness of an unequal distribution of life resources.
- A set of six questions were asked in each of three life domains: how individuals compare their work opportunities with other, their ability to provide for their children, and their living environments (see Ryff et al., 1999).

Category Construction: Low (≤1), Mid (1-2), High (>2)

**PERCEIVED DISCRIMINATION**

**Lifetime Discrimination [B1SLFEDI]:**

Items: 11 items - Self-Administered Questionnaire, Section P, Questions 1 (a - k).

(How many times in your life have you been discriminated against in each of the following ways because of such things as your race, ethnicity, gender, age, religion, physical appearance, sexual orientation, or other characteristics?)

a. “You were discouraged by a teacher or advisor from seeking higher education.”

b. “You were denied a scholarship.”

c. “You were not hired for a job.”

d. “You were not given a promotion.”

e. “You were fired.”

f. “You were prevented from renting or buying a home in the neighborhood you wanted.”

g. “You were prevented from remaining in a neighborhood because neighbors made life so uncomfortable.”

h. “You were hassled by the police.”

i. “You were denied a bank loan.”

j. “You were denied or provided inferior medical care.”

k. “You were denied or provided inferior service by a plumber, care mechanic, or other service provider.”

Coding: Each item is answered by frequency (# of times) of it happening.

Scaling: The scale was constructed by taking the number of “1 or higher” responses to the items.

**Daily Discrimination [B1SDAYDI]:**

Items: 9 items - Self-Administered Questionnaire, Section P, Questions 2 (a - i)

a. “You are treated with less courtesy than other people.”

b. “You are treated with less respect than other people.”

c. “You receive poorer service than other people at restaurants or stores.”

d. “People act as if they think you are not smart.”

e. “People act as if they are afraid of you.”

f. “People act as if they think you are dishonest.”

g. “People act as if they think you are not as good as they are.”

h. “You are called names or insulted.”

i. “You are threatened or harassed.”

Coding: 1 Often; 2 Sometimes; 3 Rarely; 4 Never.

Scaling: The scale is constructed by calculating the sum of the values of the items. Items were reverse-coded so that high scores reflect higher standing in the scale. For an item with a missing value, the mean value of completed items is imputed.

Sources: *The perceived discrimination questions were developed by one of the authors for use in a study of racial discrimination in Detroit (Williams et al., 1997). The questions were based largely on the results of previous qualitative studies of discrimination (Essed, 1991; Feagin, 1991). – from Kessler et. al. (1999) [see below]

Williams, D. R., YU, Y., Jackson, J. S., & Anderson, N. B. (1997). Racial differences in physical and mental health: Socioeconomic status, stress and discrimination. Journal of Health Psychology, 2, 335-351.

**Supplemental Material. Part B. Tables and Figures.**


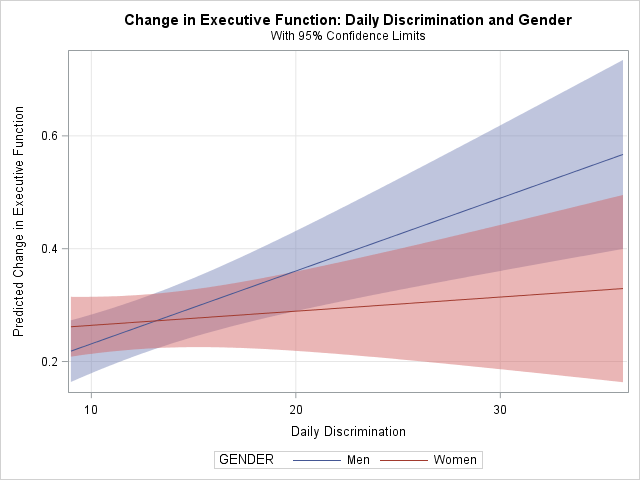


Figure 1. Predicted change in executive function over 10 years of follow-up (MIDUS2 Score - MIDUS3 Score)/follow-up time)*10 years), by daily discrimination scale with a gender interaction.

| **Supplemental Table 1. Means in episodic memory and executive function in men and women, and changes in means in age groups** | | | | | | | | | | | | | | | |
| --- | --- | --- | --- | --- | --- | --- | --- | --- | --- | --- | --- | --- | --- | --- | --- |
|  | **MIDUS 2** | | | | | | |  | **MIDUS 3** | | | | | | |
|  | **Men** | | | **Women** | | |  |  | **Men** | | | **Women** | | |  |
|  | n | Range | Mean (SD) | n | Range | Mean (SD) | P |  | n | Range | Mean (SD) | n | Range | Mean (SD) | P |
| **Episodic Memory** | | | | | | | |  |  |  |  |  |  |  |  |
| All | 493 | -2.10, 2.57 | -0.246 (0.89) | 652 | -2.50, 3.23 | 0.246 (0.89) | <.0001 |  | 414 | -2.10, 2.63 | -0.342 (0.84) | 552 | -2.50, 3.64 | 0.256 (1.04) | <.0001 |
| 40-49 | 132 | -1.88, 2.41 | -0.013 (0.72) | 169 | -1.47, 2.44 | 0.448 (0.79) | <.0001 |  | 33 | -1.44, 2.03 | 0.102 (0.89) | 60 | -1.47, 3.42 | 0.600 (1.09) | 0,028 |
| 50-59 | 147 | -1.88, 1.56 | -0.190 (0.71) | 205 | -1.66, 3.23 | 0.322 (0.90) | <.0001 |  | 105 | -1.88, 2.63 | 0.052 (0.85) | 132 | -1.44, 3.64 | 0.549 (1.00) | <.0001 |
| 60-69 | 105 | -2.10, 2.57 | -0.502 (0.74) | 137 | -1.66, 2.44 | 0.120 (0.74) | <.0001 |  | 124 | -2.10, 2.60 | -0.318 (0.72) | 145 | -1.88, 2.79 | 0.398 (0.91) | <.0001 |
| ≥70 | 73 | -2.10, 0.96 | -0.693 (0.73) | 72 | -1.66, 2.82 | -0.328 (0.56) | 0,006 |  | 119 | -1.88, 1.56 | -0.750 (0.69) | 142 | -2.50, 2.65 | -0.129 (1.04) | <.0001 |
| **Executive Functioning** | | | | | | | |  |  |  |  |  |  |  |  |
| All | 495 | -2.58, 2.01 | 0.125 (0.65) | 654 | -3.07, 1.93 | -0.001 (0.65) | 0,001 |  | 415 | -2.22, 1.97 | -0.012 (0.69) | 553 | -3.15, 1.71 | -0.188 (0.74) | 0,0002 |
| 40-49 | 132 | -0.99, 2.01 | 0.396 (0.64) | 169 | -2.01, 1.51 | 0.223 (0.62) | 0,018 |  | 33 | -1.07, 1.49 | 0.339 (0.66) | 60 | -1.06, 1.71 | 0.233 (0.63) | 0,445 |
| 50-59 | 147 | -1.38, 1.50 | 0.171 (0.58) | 206 | -1.77, 1.56 | 0.045 (0.61) | 0,051 |  | 105 | -1.41, 1.97 | 0.375 (0.67) | 132 | -1.74, 1.41 | 0.175 (0.59) | 0,016 |
| 60-69 | 105 | -1.16, 1.33 | -0.062 (0.55) | 138 | -3.07, 1.30 | -0.194 (0.63) | 0,088 |  | 124 | -1.26, 1.35 | 0.043 (0.55) | 145 | -1.66, 1.58 | -0.052 (0.63) | 0,194 |
| ≥70 | 73 | -2.58, 1.30 | -0.288 (0.63) | 72 | -2.02, 0.64 | -0.503 (0.51) | 0,025 |  | 120 | -1.58, 0.96 | -0.340 (0.55) | 143 | -3.15, 1.36 | -0.574 (0.65) | 0,002 |
| *p-values based on between group t-test by gender | | | | | | | | | | | | | | | |

| **Supplemental Table 2. Within person cognition changes from Midus 2 to 3 (B3TEM - C3TEM & B3TEF - C3TEF) Age categories based on age at MIDUS 2.** | | | | | | | | | | | | | | | | | | | |
| --- | --- | --- | --- | --- | --- | --- | --- | --- | --- | --- | --- | --- | --- | --- | --- | --- | --- | --- | --- |
|  | **ALL** | | |  | **EXCLUDING unemployed/retired** | | |  | **CAUCASIAN ONLY** | | |  | **HIGHER INCOME (>50%)** | | |  | **LOWER INCOME (≤50%)** | | |
|  | **n** | **Within person mean difference (SD)** | **p-value** |  | **n** | **Within person mean difference (SD)** | **p-value** |  | **n** | **Within person mean difference (SD)** | **p-value** |  | **n** | **Within person mean difference (SD)** | **p-value** |  | **n** | **Within person mean difference (SD)** | **p-value** |
|  |  |  |  |  |  |  |  |  |  |  |  |  |  |  |  |  |  |  |  |
| **MEN** | | | | | | | | | | | | | | | | | | | |
| **Episodic Memory** | | | | | | | | | | | | | | | | | | | |
| All | 1175 | 0.15 (0.8) | <.0001 |  | 844 | 0.11 (0.8) | 0.0001 |  | 1026 | 0.16 (0.8) | <.0001 |  | 563 | 0.11 (0.8) | 0.003 |  | 612 | 0.19 (0.9) | <.0001 |
| <40 | 82 | 0.07 (0.9) | 0.494 |  | 79 | 0.05 (0.9) | 0.609 |  | 75 | 0.10 (0.8) | 0.305 |  | 53 | -0.03 (0.9) | 0.821 |  | 29 | 0.23 (0.9) | 0.155 |
| 40-49 | 281 | -0.003 (0.9) | 0.957 |  | 256 | -0.01 (0.9) | 0.804 |  | 259 | 0.01 (0.9) | 0.856 |  | 159 | -0.01 (0.8) | 0.825 |  | 122 | 0.01 (0.9) | 0.876 |
| 50-59 | 366 | 0.16 (0.8) | 0.0003 |  | 321 | 0.14 (0.8) | 0.002 |  | 335 | 0.15 (0.8) | 0.001 |  | 208 | 0.10 (0.8) | 0.068 |  | 158 | 0.23 (0.8) | 0.001 |
| 60-69 | 261 | 0.19 (0.8) | 0.0003 |  | 147 | 0.22 (0.9) | 0.003 |  | 240 | 0.20 (0.8) | 0.0002 |  | 110 | 0.23 (0.8) | 0.003 |  | 151 | 0.15 (0.8) | 0.026 |
| ≥70 | 124 | 0.49 (0.8) | <.0001 |  | 32 | 0.59 (0.6) | <.0001 |  | 117 | 0.50 (0.8) | <.0001 |  | 33 | 0.45 (0.8) | 0.003 |  | 91 | 0.51 (0.8) | <.0001 |
| **Executive Function** | | | | | | | | | | | | | | | | | | | |
| All | 1183 | 0.26 (0.5) | <.0001 |  | 850 | 0.21 (0.4) | <.0001 |  | 1033 | 0.26 (0.5) | <.0001 |  | 565 | 0.19 (0.4) | <.0001 |  | 618 | 0.32 (0.5) | <.0001 |
| <40 | 84 | 0.21 (0.5) | <.0001 |  | 81 | 0.20 (0.5) | 0.0002 |  | 77 | 0.22 (0.5) | 0.0001 |  | 54 | 0.13 (0.5) | 0.044 |  | 30 | 0.36 (0.4) | <.0001 |
| 40-49 | 282 | 0.12 (0.4) | <.0001 |  | 266 | 0.12 (0.4) | <.0001 |  | 260 | 0.12 (0.4) | <.0001 |  | 159 | 0.09 (0.4) | 0.004 |  | 123 | 0.16 (0.4) | <.0001 |
| 50-59 | 369 | 0.24 (0.4) | <.0001 |  | 324 | 0.22 (0.4) | <.0001 |  | 338 | 0.23 (0.4) | <.0001 |  | 209 | 0.19 (0.4) | <.0001 |  | 160 | 0.31 (0.5) | <.0001 |
| 60-69 | 262 | 0.36 (0.6) | <.0001 |  | 147 | 0.27 (0.5) | <.0001 |  | 241 | 0.36 (0.6) | <.0001 |  | 110 | 0.33 (0.5) | <.0001 |  | 152 | 0.38 (0.6) | <.0001 |
| ≥70 | 124 | 0.47 (0.5) | <.0001 |  | 32 | 0.53 (0.4) | <.0001 |  | 117 | 0.48 (0.5) | <.0001 |  | 33 | 0.45 (0.4) | <.0001 |  | 91 | 0.48 (0.5) | <.0001 |
| **WOMEN** | | | | | | | | | | | | | | | | | | | |
| **Episodic Memory** | | | | | | | | | | | | | | | | | | | |
| All | 1523 | 0.09 (1.0) | 0.0002 |  | 851 | 0.03 (0.9) | 0.391 |  | 1253 | 0.12 (0.9) | <.0001 |  | 610 | 0.01 (1.0) | 0.714 |  | 913 | 0.15 (1.0) | <.0001 |
| <40 | 119 | 0.06 (0.9) | 0.480 |  | 95 | 0.04 (0.9) | 0.635 |  | 105 | 0.12 (0.9) | 0.161 |  | 55 | 0.12 (1.0) | 0.359 |  | 64 | 0.01 (0.9) | 0.961 |
| 40-49 | 354 | -0.07 (0.9) | 0.142 |  | 291 | -0.07 (0.9) | 0.185 |  | 325 | -0.07 (0.9) | 0.147 |  | 201 | -0.18 (0.9) | 0.008 |  | 153 | 0.07 (0.8) | 0.331 |
| 50-59 | 406 | 0.07 (0.9) | 0.130 |  | 305 | 0.00 (0.9) | 0.997 |  | 367 | 0.05 (0.9) | 0.318 |  | 216 | 0.06 (0.9) | 0.317 |  | 190 | 0.08 (0.9) | 0.255 |
| 60-69 | 335 | 0.22 (1.0) | <.0001 |  | 138 | 0.24 (1.0) | 0.006 |  | 301 | 0.23 (1.0) | <.0001 |  | 107 | 0.16 (1.0) | 0.084 |  | 228 | 0.24 (1.0) | 0.0003 |
| ≥70 | 168 | 0.44 (1.0) | <.0001 |  | 22 | 0.26 (1.3) | 0.361 |  | 156 | 0.44 (0.6) | <.0001 |  | 31 | 0.20 (1.1) | 0.324 |  | 137 | 0.50 (0.9) | <.0001 |
| **Executive Function** | | | | | | | | | | | | | | | | | | | |
| All | 1527 | 0.25 (0.5) | <.0001 |  | 853 | 0.19 (0.5) | <.0001 |  | 1257 | 0.26 (0.5) | <.0001 |  | 611 | 0.24 (0.5) | <.0001 |  | 916 | 0.25 (0.5) | <.0001 |
| <40 | 119 | 0.12 (0.4) | 0.0002 |  | 95 | 0.09 (0.4) | 0.051 |  | 105 | 0.14 (0.4) | 0.002 |  | 55 | 0.13 (0.4) | 0.012 |  | 64 | 0.12 (0.5) | 0.054 |
| 40-49 | 353 | 0.13 (0.4) | <.0001 |  | 290 | 0.11 (0.4) | <.0001 |  | 324 | 0.13 (0.4) | <.0001 |  | 200 | 0.14 (0.4) | <.0001 |  | 153 | 0.13 (0.5) | 0.001 |
| 50-59 | 409 | 0.25 (0.5) | <.0001 |  | 307 | 0.22 (0.5) | <.0001 |  | 370 | 0.26 (0.5) | <.0001 |  | 217 | 0.26 (0.5) | <.0001 |  | 192 | 0.24 (0.5) | <.0001 |
| 60-69 | 336 | 0.34 (0.5) | <.0001 |  | 139 | 0.32 (0.5) | <.0001 |  | 302 | 0.36 (0.5) | <.0001 |  | 107 | 0.37 (0.5) | <.0001 |  | 229 | 0.33 (0.5) | <.0001 |
| ≥70 | 169 | 0.44 (0.6) | <.0001 |  | 22 | 0.41 (0.5) | 0.001 |  | 156 | 0.44 (0.6) | <.0001 |  | 32 | 0.50 (0.5) | <.0001 |  | 137 | 0.43 (0.6) | <.0001 |

| **Supplemental Table 3. SES/demographic variable descriptions (N (%) or Mean (SD)), and how they predict cognitive change.** | | | | | | | | | | | | | | | | |
| --- | --- | --- | --- | --- | --- | --- | --- | --- | --- | --- | --- | --- | --- | --- | --- | --- |
| Outcome: MIDUS2 Cognition - MIDUS3 Cognition. Meaning higher score represents more decline, negative score means less decline/incline from baseline cognition score | | | | | | | | | | | | | | | | |
|  |  |  |  |  | **Men** | | | | | | **Women** | | | | | |
| Variables | Range | Men | Women |  | Executive Function | | | Episodic Memory | | | Executive Function | | | Episodic Memory | | |
|  |  | N (%) or Mean (SD) | |  | β (SE) | P-value | Adj R^2^ | β (SE) | P-value | Adj R^2^ | β (SE) | P-value | Adj R^2^ | β (SE) | P-value | Adj R^2^ |
| **SES/Demographics** |  |  |  |  | **n=985** | | | **n=980** | | | **n=1271** | | | **n=1235** | | |
| Age at MIDUS 2 (Baseline) | 33-83 | 55.2 (10.9) | 55.2 (11.4) |  | 0.01 (0.002) | <.0001 | 0.127 | 0.03 (0.003) | <.0001 | 0.35 | 0.02 (0.002) | <.0001 | 0.17 | 0.02 (0.003) | <.0001 | 0.26 |
| Baseline Cognition Score |  |  |  |  | 0.23 (0.03) | <.0001 |  | 0.68 (0.03) | <.0001 |  | 0.33 (0.03) | <.0001 |  | 0.57 (0.03) | <.0001 |  |
| Hispanic (vs White) |  | 28 (2.4) | 45 (2.9) |  | 0.03 (0.11) | 0.798 |  | 0.03 (0.17) | 0.857 |  | 0.05 (0.08) | 0.539 |  | 0.22 (0.15) | 0.128 |  |
| Other (vs White) |  | 57 (4.8) | 81 (5.3) |  | 0.14 (0.07) | 0.067 |  | 0.01 (0.12) | 0.907 |  | 0.06 (0.07) | 0.378 |  | 0.18 (0.12) | 0.141 |  |
| Some college or more (vs High School or Less Education) |  | 853 (71.9) | 920 (60.0) |  | -0.06 (0.04) | 0.076 |  | -0.10 (0.06) | 0.096 |  | -0.03 (0.03) | 0.424 |  | -0.06 (0.06) | 0.299 |  |
| Living with partner (vs not) |  | 215 (18.1) | 459 (29.9) |  | 0.06 (0.04) | 0.135 |  | 0.004 (0.06) | 0.945 |  | 0.03 (0.03) | 0.338 |  | -0.03 (0.06) | 0.559 |  |
| Income (per 100% above FPL) | 0-25 | 5.6 (4.1) | 4.5 (3.7) |  | -0.01 (0.004) | 0.158 |  | -0.02 (0.006) | 0.008 |  | -0.005 (0.004) | 0.214 |  | -0.02 (0.007) | 0.023 |  |
| Unemployed (vs employed) |  | 26 (2.2) | 180 (11.7) |  | 0.13 (0.11) | 0.235 |  | 0.27 (0.17) | 0.111 |  | 0.08 (0.04) | 0.069 |  | 0.03 (0.08) | 0.735 |  |
| Retired (vs employed) |  | 245 (20.6) | 348 (22.7) |  | 0.19 (0.05) | <.0001 |  | 0.001 (0.07) | 0.988 |  | 0.09 (0.04) | 0.019 |  | 0.05 (0.07) | 0.502 |  |
| Physical Health | 1-5 | 2.3 (0.9) | 2.4 (0.9) |  | 0.02 (0.02) | 0.181 |  | 0.02 (0.03) | 0.591 |  | 0.02 (0.02) | 0.212 |  | 0.10 (0.03) | 0.001 |  |
| Vigorous Physical Activity (vs none) | 0-13.5 | 7.8 (5.1) | 5.3 (5.1) |  | -0.004 (0.003) | 0.180 |  | 0.00 (0.005) | 0.919 |  | -0.005 (0.003) | 0.100 |  | -0.003 (0.005) | 0.571 |  |
| Depression (continuous scale) | 0-7 | 0.3 (1.3) | 0.7 (1.9) |  | 0.005 (0.01) | 0.702 |  | 0.01 (0.02) | 0.541 |  | -0.004 (0.01) | 0.652 |  | -0.00 (0.01) | 0.949 |  |
| Anxiety (continuous scale) | 0-10 | 0.04 (0.5) | 0.15 (0.9) |  | 0.02 (0.03) | 0.499 |  | -0.06 (0.05) | 0.253 |  | 0.02 (0.02) | 0.313 |  | 0.08 (0.03) | 0.013 |  |

**Figure 1. Changes in executive function**


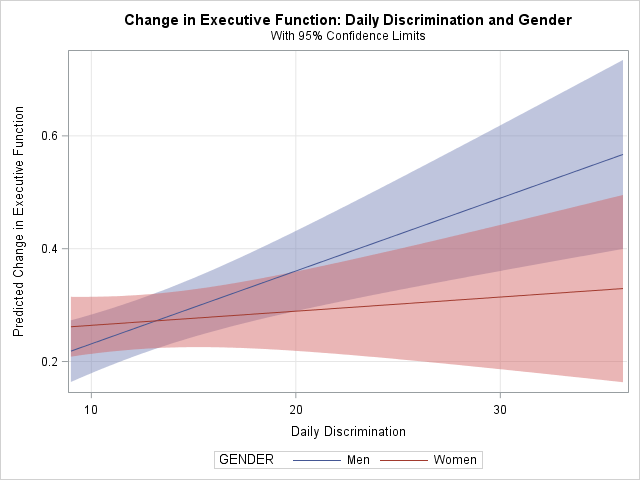


|  |
| --- |

| **Supplemental table 4. Sensitivity Analyses: Stress/strain variables and predicting change in cognition, Caucasian's only**  Outcome: (MIDUS2 Cognition - MIDUS3 Cognition)/follow-up time)*10. Higher score represents more decline, negative score means less decline from baseline cognition score | | | | | | | | | | | | |
| --- | --- | --- | --- | --- | --- | --- | --- | --- | --- | --- | --- | --- |
|  |  | **Executive Function** | | | | |  | **Episodic Memory** | | | | |
|  |  | **MEN** | |  | **WOMEN** | |  | **MEN** | |  | **WOMEN** | |
| **Scales** |  | **SA: CAUCASIAN ONLY, n=742-785** | |  | **SA: CAUCASIAN ONLY, n=776-1171** | |  | **SA: CAUCASIAN ONLY, n=738-794** | |  | **SA: CAUCASIAN ONLY, n=** | |
|  |  | β (SE) | P-value |  | β (SE) | P-value |  | β (SE) | P-value |  | β (SE) | P-value |
| **Family & Marital Level Stress** | | | |  |  |  |  |  |  |  |  |  |
| Marital risk | 0 | ref | |  | ref | |  | ref | |  | ref | |
|  | 1 | -0.06 (0.05) | 0.22 |  | 0.01 (0.05) | 0.83 |  | -0.15 (0.07) | 0.05 |  | -0.08 (0.09) | 0.41 |
|  | 2 | -0.01 (0.04) | 0.81 |  | 0.06 (0.04) | 0.08 |  | 0.16 (0.07) | 0.01 |  | 0.04 (0.07) | 0.57 |
| Family strain | 0 | ref | |  | ref | |  | ref | |  | ref | |
|  | 1 | -0.03 (0.07) | 0.66 |  | -0.03 (0.07) | 0.67 |  | 0.07 (0.10) | 0.50 |  | 0.12 (0.13) | 0.35 |
|  | 2 | 0.04 (0.07) | 0.58 |  | 0.003 (0.07) | 0.97 |  | 0.08 (0.11) | 0.48 |  | 0.15 (0.13) | 0.25 |
| Spouse/ partner strain | 0 | ref | |  | ref | |  | ref | |  | ref | |
|  | 1 | -0.03 (0.14) | 0.82 |  | -0.02 (0.08) | 0.84 |  | 0.18 (0.22) | 0.42 |  | -0.11 (0.16) | 0.49 |
|  | 2 | -0.06 (0.14) | 0.64 |  | -0.01 (0.08) | 0.94 |  | 0.24 (0.22) | 0.28 |  | -0.07 (0.16) | 0.69 |
| **Work Level Stress** |  |  |  |  |  |  |  |  |  |  |  |  |
| Perceived Inequality at work | 0 | ref | |  | ref | |  | ref | |  | ref | |
|  | 1 | 0.02 (0.04) | 0.56 |  | -0.01 (0.04) | 0.90 |  | 0.09 (0.07) | 0.16 |  | -0.04 (0.08) | 0.62 |
|  | 2 | 0.05 (0.06) | 0.32 |  | 0.03 (0.06) | 0.55 |  | 0.13 (0.09) | 0.17 |  | 0.08 (0.11) | 0.49 |
| Chronic job discrimination | 0 | Ref | |  | Ref | |  | Ref | |  | ref | |
|  | 1 | 0.02 (0.04) | 0.53 |  | 0.03 (0.04) | 0.45 |  | 0.06 (0.06) | 0.32 |  | 0.001 (0.07) | 0.99 |
| **Society Level Stress** |  |  |  |  |  |  |  |  |  |  |  |  |
| Perceived Inequality in family | 0 | ref | |  | ref | |  | ref | |  | ref | |
|  | 1 | -0.03 (0.04) | 0.51 |  | 0.05 (0.04) | 0.23 |  | 0.05 (0.07) | 0.48 |  | 0.04 (0.07) | 0.52 |
|  | 2 | 0.09 (0.06) | 0.15 |  | 0.09 (0.05) | 0.09 |  | 0.17 (0.11) | 0.11 |  | 0.30 (0.09) | 0.001 |
| Lifetime |  | 0.002 (0.01) | 0.86 |  | -0.01 (0.01) | 0.47 |  | 0.02 (0.02) | 0.23 |  | -0.03 (0.02) | 0.10 |
| Daily |  | 0.01 (0.004) | 0.01 |  | 0.001 (0.004) | 0.77 |  | 0.01 (0.01) | 0.07 |  | 0.003 (0.01) | 0.72 |
| *All models control for age at MIDUS 2, baseline cognition score, race/ethnicity (White, Hispanic, other), education (some college or more vs high school of less), living with partner, income (per 100% above FPL), unemployed (vs employed), retired (vs employed), physical health (self-reported), vigorous physical activity, depression (continuous scale), anxiety (continuous scale) | | | | | | | | | | | | |

| **Supplemental Table 5. Stress/strain variables and predicting episodic memory and executive function at MIDUS 2 (cross-sectional)** | | | | | | | | | | | | |
| --- | --- | --- | --- | --- | --- | --- | --- | --- | --- | --- | --- | --- |
| Outcome: MIDUS2 Cognition. Higher score represents better baseline cognitive score, lower score represents worse baseline cognitive score | | | | | | | | | | | | |
|  |  | **Episodic Memory** | | | | |  | **Executive Function** | | | | |
| **Scales** | **Tertile** | **Men (n=785-984)** | |  | **Women (n=818-1234)** | |  | **Men (n=785-984)** | |  | **Women (n=818-1234)** | |
|  |  | β (SE) | P-value |  | β (SE) | P-value |  | β (SE) | P-value |  | β (SE) | P-value |
| **Family & Marital Level Stress** | | | | | | | | | | | | |
| Perceived Inequality of family | 0 | ref | |  | ref | |  | ref | |  | ref | |
|  | 1 | -0.11 (0.06) | 0.09 |  | 0.02 (0.07) | 0.71 |  | -0.004 (0.05) | 0.93 |  | 0.005 (0.04) | 0.90 |
|  | 2 | -0.30 (0.10) | 0.003 |  | 0.04 (0.09) | 0.65 |  | 0.02 (0.07) | 0.74 |  | -0.08 (0.05) | 0.14 |
| Marital risk | 0 | ref | |  | ref | |  | ref | |  | ref | |
|  | 1 | -0.05 (0.08) | 0.55 |  | 0.13 (0.09) | 0.15 |  | -0.10 (0.05) | 0.08 |  | 0.04 (0.05) | 0.50 |
|  | 2 | 0.02 (0.07) | 0.76 |  | 0.05 (0.07) | 0.48 |  | -0.12 (0.05) | 0.01 |  | 0.05 (0.04) | 0.24 |
| Family strain | 0 | ref | |  | ref | |  | ref | |  | ref | |
|  | 1 | 0.16 (0.10) | 0.12 |  | 0.12 (0.12) | 0.33 |  | 0.04 (0.07) | 0.60 |  | 0.06 (0.07) | 0.37 |
|  | 2 | 0.09 (0.11) | 0.40 |  | 0.06 (0.12) | 0.60 |  | -0.00 (0.08) | 1.00 |  | -0.001 (0.07) | 0.99 |
| Spouse/ partner strain | 0 | ref | |  | ref | |  | ref | |  | ref | |
|  | 1 | 0.02 (0.23) | 0.95 |  | -0.08 (0.16) | 0.61 |  | 0.09 (0.17) | 0.61 |  | 0.02 (0.09) | 0.84 |
|  | 2 | -0.01 (0.23) | 0.96 |  | -0.01 (0.16) | 0.93 |  | 0.07 (0.17) | 0.67 |  | -0.01 (0.09) | 0.88 |
| **Work Level Stress** | | | | | | | | | | | |  |
| Perceived Inequality at work | 0 | ref | |  | ref | |  | ref | |  | ref | |
|  | 1 | -0.07 (0.07) | 0.29 |  | -0.06 (0.07) | 0.39 |  | -0.12 (0.05) | 0.01 |  | -0.10 (0.05) | 0.03 |
|  | 2 | -0.23 (0.09) | 0.01 |  | -0.04 (0.10) | 0.67 |  | -0.23 (0.07) | 0.0006 |  | -0.15 (0.06) | 0.02 |
| Chronic job discrimination | 0 | ref | |  | ref | |  | ref | |  | ref | |
|  | 1 | 0.01 (0.05) | 0.82 |  | 0.06 (0.04) | 0.11 |  | -0.04 (0.06) | 0.55 |  | 0.001 (0.07) | 0.99 |
| **Society Level Stress** | | | | | | | | | | | | |
| Lifetime discrimination | cont' | -0.03 (0.02) | 0.18 |  | 0.02 (0.02) | 0.40 |  | -0.04 (0.01) | 0.007 |  | 0.01 (0.01) | 0.17 |
| Daily discrimination | cont' | -0.01 (0.01) | 0.02 |  | -0.005 (0.01) | 0.48 |  | -0.02 (0.002) | <.0001 |  | -0.01 (0.004) | 0.002 |
| *All models control for age at MIDUS 2, baseline cognition score, race/ethnicity (White, Hispanic, other), education (some college or more vs high school of less), living with partner, income (per 100% above FPL), unemployed (vs employed), retired (vs employed), physical health (self-reported), vigorous physical activity, depression (continuous scale), anxiety (continuous scale) | | | | | | | | | | | | |

| **Supplemental Table 6. SES/demographic variable descriptions (N (%) or Mean (SD)), and how they predict cognitive change.** | | | | | | | | | | | | | | | | |
| --- | --- | --- | --- | --- | --- | --- | --- | --- | --- | --- | --- | --- | --- | --- | --- | --- |
| Outcome: MIDUS2 Cognition - MIDUS3 Cognition. Meaning higher score represents more decline, negative score means less decline/incline from baseline cognition score | | | | | | | | | | | | | | | | |
|  |  |  |  |  | **Men** | | | | | | **Women** | | | | | |
| Variables | Range | Men | Women |  | Executive Function | | | Episodic Memory | | | Executive Function | | | Episodic Memory | | |
|  |  | N (%) or Mean (SD) | |  | β (SE) | P-value | Adj R^2^ | β (SE) | P-value | Adj R^2^ | β (SE) | P-value | Adj R^2^ | β (SE) | P-value | Adj R^2^ |
| **SES/Demographics** |  |  |  |  | **n=985** | | | **n=980** | | | **n=1271** | | | **n=1235** | | |
| Age at MIDUS 2 (Baseline) | 33-83 | 55.2 (10.9) | 55.2 (11.4) |  | 0.01 (0.002) | <.0001 | 0.127 | 0.03 (0.003) | <.0001 | 0.35 | 0.02 (0.002) | <.0001 | 0.17 | 0.02 (0.003) | <.0001 | 0.26 |
| Baseline Cognition Score |  |  |  |  | 0.23 (0.03) | <.0001 |  | 0.68 (0.03) | <.0001 |  | 0.33 (0.03) | <.0001 |  | 0.57 (0.03) | <.0001 |  |
| Hispanic (vs White) |  | 28 (2.4) | 45 (2.9) |  | 0.03 (0.11) | 0.798 |  | 0.03 (0.17) | 0.857 |  | 0.05 (0.08) | 0.539 |  | 0.22 (0.15) | 0.128 |  |
| Other (vs White) |  | 57 (4.8) | 81 (5.3) |  | 0.14 (0.07) | 0.067 |  | 0.01 (0.12) | 0.907 |  | 0.06 (0.07) | 0.378 |  | 0.18 (0.12) | 0.141 |  |
| Some college or more (vs High School or Less Education) |  | 853 (71.9) | 920 (60.0) |  | -0.06 (0.04) | 0.076 |  | -0.10 (0.06) | 0.096 |  | -0.03 (0.03) | 0.424 |  | -0.06 (0.06) | 0.299 |  |
| Living with partner (vs not) |  | 215 (18.1) | 459 (29.9) |  | 0.06 (0.04) | 0.135 |  | 0.004 (0.06) | 0.945 |  | 0.03 (0.03) | 0.338 |  | -0.03 (0.06) | 0.559 |  |
| Income (per 100% above FPL) | 0-25 | 5.6 (4.1) | 4.5 (3.7) |  | -0.01 (0.004) | 0.158 |  | -0.02 (0.006) | 0.008 |  | -0.005 (0.004) | 0.214 |  | -0.02 (0.007) | 0.023 |  |
| Unemployed (vs employed) |  | 26 (2.2) | 180 (11.7) |  | 0.13 (0.11) | 0.235 |  | 0.27 (0.17) | 0.111 |  | 0.08 (0.04) | 0.069 |  | 0.03 (0.08) | 0.735 |  |
| Retired (vs employed) |  | 245 (20.6) | 348 (22.7) |  | 0.19 (0.05) | <.0001 |  | 0.001 (0.07) | 0.988 |  | 0.09 (0.04) | 0.019 |  | 0.05 (0.07) | 0.502 |  |
| Physical Health | 1-5 | 2.3 (0.9) | 2.4 (0.9) |  | 0.02 (0.02) | 0.181 |  | 0.02 (0.03) | 0.591 |  | 0.02 (0.02) | 0.212 |  | 0.10 (0.03) | 0.001 |  |
| Vigorous Physical Activity (vs none) | 0-13.5 | 7.8 (5.1) | 5.3 (5.1) |  | -0.004 (0.003) | 0.180 |  | 0.00 (0.005) | 0.919 |  | -0.005 (0.003) | 0.100 |  | -0.003 (0.005) | 0.571 |  |
| Depression (continuous scale) | 0-7 | 0.3 (1.3) | 0.7 (1.9) |  | 0.005 (0.01) | 0.702 |  | 0.01 (0.02) | 0.541 |  | -0.004 (0.01) | 0.652 |  | -0.00 (0.01) | 0.949 |  |
| Anxiety (continuous scale) | 0-10 | 0.04 (0.5) | 0.15 (0.9) |  | 0.02 (0.03) | 0.499 |  | -0.06 (0.05) | 0.253 |  | 0.02 (0.02) | 0.313 |  | 0.08 (0.03) | 0.013 |  |

| **Supplemental Table 7. Spearman Correlation Coefficients between discrimination measures and SES/other measures** | | | | | | | | | |
| --- | --- | --- | --- | --- | --- | --- | --- | --- | --- |
|  | **MEN, N = 979** | | | |  | **WOMEN, N = 1230** | | | |
| **SES/Other Measures** | **Lifetime Discrimination** | | **Daily Discrimination** | |  | **Lifetime Discrimination** | | **Daily Discrimination** | |
|  | Correlation Coefficient | P-value | Correlation Coefficient | P-value |  | Correlation Coefficient | P-value | Correlation Coefficient | P-value |
| Daily Discrimination | -0.05 | 0.10 | 1.00 |  |  | -0.06 | 0.05 | 1.00 |  |
| Age at MIDUS 2 | 0.12 | 0.0002 | -0.10 | 0.002 |  | 0.11 | 0.0002 | -0.18 | <.0001 |
| Hispanic | -0.02 | 0.48 | 0.06 | 0.08 |  | -0.03 | 0.29 | 0.03 | 0.36 |
| Other non-Caucasian Race | 0.03 | 0.29 | 0.12 | 0.0002 |  | 0.01 | 0.71 | 0.08 | 0.006 |
| Some College/More | -0.01 | 0.71 | -0.09 | 0.01 |  | -0.01 | 0.72 | 0.03 | 0.36 |
| Unemployed | -0.02 | 0.47 | 0.01 | 0.73 |  | 0.00 | 0.91 | 0.00 | 0.97 |
| Retired/Other | 0.07 | 0.03 | -0.07 | 0.03 |  | 0.07 | 0.02 | -0.11 | 0.0001 |
| Married or living w/ someone | -0.04 | 0.24 | 0.06 | 0.05 |  | -0.03 | 0.23 | 0.14 | <.0001 |
| Income | -0.04 | 0.18 | -0.02 | 0.59 |  | -0.10 | 0.001 | 0.02 | 0.54 |
| Physical health | 0.06 | 0.06 | 0.07 | 0.03 |  | 0.01 | 0.62 | 0.18 | <.0001 |
| Times/ month vigorous activity | -0.05 | 0.09 | 0.05 | 0.11 |  | 0.01 | 0.86 | -0.02 | 0.44 |
| Depressed Affect + Anhedonia (cont.) | 0.01 | 0.71 | 0.00 | 0.95 |  | -0.06 | 0.05 | 0.12 | <.0001 |
| Anxiety Disorder (cont.) | -0.01 | 0.65 | -0.04 | 0.23 |  | 0.00 | 0.88 | 0.05 | 0.10 |

| **Supplemental Table 8. Sensitivity Analyses: Stress/strain variables and predicting change in episodic memory change** | | | | | | | | | | | | | | | | | | | | | | |
| --- | --- | --- | --- | --- | --- | --- | --- | --- | --- | --- | --- | --- | --- | --- | --- | --- | --- | --- | --- | --- | --- | --- |
| Outcome: (MIDUS2 Cognition - MIDUS3 Cognition)/follow-up time)*10. Higher score represents more decline, negative score means less decline from baseline cognition score | | | | | | | | | | | | | | | | | | | | | | |
|  |  | **Episodic Memory** | | | | | | | | | | | | | | | | | | | | |
|  |  | **MEN** | | | | | | | |  | **WOMEN** | | | | | | | | | | | |
| **Scales** |  | **SA: CAUCASIAN ONLY, n=738-794** | |  | **SA: HIGHER INCOME (>50%), n=497-541** | |  | **SA: LOWER INCOME (≤50%), n=347-430** | |  | **SA: CAUCASIAN ONLY, n=** | |  | | **SA: HIGHER INCOME (>50%), n= 596** | | |  | | **SA: LOWER INCOME (≤50%), n=387-672** | | |
|  |  | β (SE) | P-value |  | β (SE) | P-value |  | β (SE) | P-value |  | β (SE) | P-value | |  | | β (SE) | P-value | |  | | β (SE) | P-value |
| **Family & Marital Level Stress** | | | | | | | | | | | | | | | | | | | | | | |
| Perceived Inequality in family | 0 | ref | |  | ref | |  | ref | |  | ref | |  | | ref | | |  | | ref | | |
|  | 1 | 0.05 (0.07) | 0.48 |  | 0.08 (0.08) | 0.30 |  | -0.05 (0.10) | 0.62 |  | 0.04 (0.07) | 0.52 | |  | | 0.03 (0.09) | 0.76 | |  | | 0.07 (0.10) | 0.51 |
|  | 2 | 0.17 (0.11) | 0.11 |  | 0.51 (0.15) | 0.001 |  | -0.14 (0.13) | 0.29 |  | 0.30 (0.09) | 0.001 | |  | | 0.18 (0.14) | 0.21 | |  | | 0.29 (0.12) | 0.01 |
| Marital risk | 0 | ref | |  | ref | |  | ref | |  | ref | |  | | ref | | |  | | ref | | |
|  | 1 | -0.15 (0.07) | 0.05 |  | -0.12 (0.10) | 0.20 |  | -0.16 (0.11) | 0.15 |  | -0.08 (0.09) | 0.41 | |  | | -0.09 (0.11) | 0.42 | |  | | -0.02 (0.15) | 0.89 |
|  | 2 | 0.16 (0.07) | 0.01 |  | 0.26 (0.08) | 0.002 |  | -0.03 (0.09) | 0.74 |  | 0.04 (0.07) | 0.57 | |  | | 0.01 (0.09) | 0.90 | |  | | -0.01 (0.10) | 0.95 |
| Family strain | 0 | ref | |  | ref | |  | ref | |  | ref | |  | | ref | | |  | | ref | | |
|  | 1 | 0.07 (0.10) | 0.50 |  | 0.12 (0.14) | 0.41 |  | 0.04 (0.14) | 0.76 |  | 0.12 (0.13) | 0.35 | |  | | 0.07 (0.20) | 0.72 | |  | | 0.05 (0.15) | 0.73 |
|  | 2 | 0.08 (0.11) | 0.48 |  | 0.11 (0.15) | 0.44 |  | 0.08 (0.14) | 0.56 |  | 0.15 (0.13) | 0.25 | |  | | 0.06 (0.20) | 0.75 | |  | | 0.14 (0.16) | 0.36 |
| Spouse/ partner strain | 0 | ref | |  | ref | |  | ref | |  | ref | |  | | ref | | |  | | ref | | |
|  | 1 | 0.18 (0.22) | 0.42 |  | 0.57 (0.36) | 0.11 |  | 0.02 (0.27) | 0.95 |  | -0.11 (0.16) | 0.49 | |  | | -0.42 (0.22) | 0.06 | |  | | 0.16 (0.23) | 0.49 |
|  | 2 | 0.24 (0.22) | 0.28 |  | 0.65 (0.36) | 0.07 |  | 0.05 (0.27) | 0.84 |  | -0.07 (0.16) | 0.69 | |  | | -0.35 (0.22) | 0.12 | |  | | 0.18 (0.23) | 0.43 |
| **Work Level Stress** | | | | | | | | | | | | | | | | | | | | | | |
| Perceived Inequality at work | 0 | ref | |  | ref | |  | ref | |  | ref | |  | | ref | | |  | | ref | | |
|  | 1 | 0.09 (0.07) | 0.16 |  | 0.16 (0.08) | 0.04 |  | -0.06 (0.11) | 0.56 |  | -0.04 (0.08) | 0.62 | |  | | -0.11 (0.10) | 0.31 | |  | | -0.05 (0.12) | 0.67 |
|  | 2 | 0.13 (0.09) | 0.17 |  | 0.22 (0.12) | 0.07 |  | -0.07 (0.13) | 0.61 |  | 0.08 (0.11) | 0.49 | |  | | -0.11 (0.16) | 0.48 | |  | | 0.13 (0.15) | 0.40 |
| Chronic job discrimination** | 0 | ref | |  | ref | |  | ref | |  | ref | |  | | ref | | |  | | ref | | |
|  | 1 | -0.05 (0.07) | 0.52 |  | 0.09 (0.09) | 0.30 |  | -0.28 (0.11) | 0.008 |  | 0.02 (0.08) | 0.77 | |  | | 0.04 (0.10) | 0.72 | |  | | 0.07 (0.12) | 0.52 |
|  | 2 | -0.08 (0.07) | 0.27 |  | 0.05 (0.09) | 0.62 |  | -0.19 (0.10) | 0.06 |  | -0.04 (0.09) | 0.658 | |  | | -0.00 (0.12) | 0.97 | |  | | -0.00 (0.12) | 0.98 |
| **Society Level Stress** | | | | | | | | | | | | | | | | | | | | | | |
| Lifetime discrimination |  | 0.02 (0.02) | 0.23 |  | 0.04 (0.03) | 0.18 |  | -0.004 (0.02) | 0.87 |  | -0.03 (0.02) | 0.10 | |  | | -0.05 (0.03) | 0.09 | |  | | -0.01 (0.03) | 0.82 |
| Daily discrimination |  | 0.01 (0.01) | 0.07 |  | 0.01 (0.01) | 0.42 |  | 0.01 (0.01) | 0.20 |  | 0.003 (0.01) | 0.72 | |  | | 0.001 (0.01) | 0.96 | |  | | 0.01 (0.01) | 0.13 |
| *All models control for age at MIDUS 2, baseline cognition score, race/ethnicity (White, Hispanic, other), education (some college or more vs high school of less), living with partner, income (per 100% above FPL), unemployed (vs employed), retired (vs employed), physical health (self-reported), vigorous physical activity, depression (continuous scale), anxiety (continuous scale) | | | | | | | | | | | | | | | | | | | | | | |
| **Note on chronic job discrimination: higher score represents less frequent reported discrimination (see variable questionnaire/construct) | | | | | | | | | | | | | | | | | | | | | | |

| **Supplemental Table 9. Sensitivity Analyses: Stress/strain variables and predicting change in executive function change** | | | | | | | | | | | | | | | | | | | | | | |
| --- | --- | --- | --- | --- | --- | --- | --- | --- | --- | --- | --- | --- | --- | --- | --- | --- | --- | --- | --- | --- | --- | --- |
| Outcome: (MIDUS2 Cognition - MIDUS3 Cognition)/follow-up time)*10. Higher score represents more decline, negative score means less decline from baseline cognition score | | | | | | | | | | | | | | | | | | | | | | |
|  |  | **Executive Function** | | | | | | | | | | | | | | | | | | | | |
|  |  | **MEN** | | | | | | | | | | |  | | **WOMEN** | | | | | | | |
| **Scales** |  | **SA: CAUCASIAN ONLY, n=742-785** | | |  | | **SA: HIGHER INCOME (>50%), n=453-503** | |  | **SA: LOWER INCOME (≤50%), n=297-350** | | |  | | **SA: CAUCASIAN ONLY, n=776-1171** | |  | **SA: HIGHER INCOME (>50%), n= 452-601** | |  | **SA: LOWER INCOME (≤50%), n=412-678** | |
|  |  | β (SE) | P-value |  | | β (SE) | | P-value |  | β (SE) | P-value |  | | β (SE) | | P-value |  | β (SE) | P-value |  | β (SE) | P-value |
| **Family & Marital Level Stress** | | | | | | | | | | | | | | | | | | | | | | |
| Perceived Inequality in family | 0 | ref | | |  | | ref | |  | ref | | |  | | ref | |  | ref | |  | ref | |
|  | 1 | -0.03 (0.04) | 0.51 |  | | -0.04 (0.04) | | 0.41 |  | -0.01 (0.07) | 0.89 |  | | 0.05 (0.04) | | 0.23 |  | 0.09 (0.05) | 0.06 |  | -0.03 (0.06) | 0.64 |
|  | 2 | 0.09 (0.06) | 0.15 |  | | 0.09 (0.08) | | 0.30 |  | 0.05 (0.10) | 0.62 |  | | 0.09 (0.05) | | 0.09 |  | 0.10 (0.07) | 0.16 |  | 0.04 (0.07) | 0.60 |
| Marital risk | 0 | ref | | |  | | ref | |  | ref | | |  | | ref | |  | ref | |  | ref | |
|  | 1 | -0.06 (0.05) | 0.22 |  | | -0.04 (0.05) | | 0.45 |  | -0.01 (0.08) | 0.89 |  | | 0.01 (0.05) | | 0.83 |  | -0.01 (0.05) | 0.85 |  | 0.03 (0.08) | 0.72 |
|  | 2 | -0.01 (0.04) | 0.81 |  | | -0.03 (0.05) | | 0.53 |  | 0.08 (0.07) | 0.28 |  | | 0.06 (0.04) | | 0.08 |  | 0.03 (0.04) | 0.54 |  | 0.06 (0.06) | 0.25 |
| Family strain | 0 | ref | | |  | | ref | |  | ref | | |  | | ref | |  | ref | |  | ref | |
|  | 1 | -0.03 (0.07) | 0.66 |  | | -0.01 (0.08) | | 0.94 |  | -0.04 (0.10) | 0.71 |  | | -0.03 (0.07) | | 0.67 |  | -0.03 (0.10) | 0.76 |  | -0.05 (0.09) | 0.56 |
|  | 2 | 0.04 (0.07) | 0.58 |  | | 0.03 (0.08) | | 0.75 |  | 0.05 (0.11) | 0.66 |  | | 0.003 (0.07) | | 0.97 |  | 0.06 (0.10) | 0.54 |  | -0.04 (0.09) | 0.63 |
| Spouse/ partner strain | 0 | ref | | |  | | ref | |  | ref | | |  | | ref | |  | ref | |  | ref | |
|  | 1 | -0.03 (0.14) | 0.82 |  | | -0.01 (0.19) | | 0.97 |  | -0.03 (0.20) | 0.89 |  | | -0.02 (0.08) | | 0.84 |  | -0.08 (0.11) | 0.46 |  | 0.06 (0.12) | 0.61 |
|  | 2 | -0.06 (0.14) | 0.64 |  | | -0.06 (0.19) | | 0.76 |  | 0.02 (0.20) | 0.91 |  | | -0.01 (0.08) | | 0.94 |  | -0.08 (0.11) | 0.48 |  | 0.09 (0.12) | 0.49 |
| **Work Level Stress** | | | | | | | | | | | | | | | | | | | | | | |
| Perceived Inequality at work | 0 | ref | | |  | | ref | |  | ref | | |  | | ref | |  | ref | |  | ref | |
|  | 1 | 0.02 (0.04) | 0.56 |  | | 0.01 (0.04) | | 0.78 |  | 0.08 (0.07) | 0.28 |  | | -0.01 (0.04) | | 0.90 |  | 0.01 (0.05) | 0.79 |  | 0.002 (0.07) | 0.97 |
|  | 2 | 0.05 (0.06) | 0.32 |  | | 0.03 (0.04) | | 0.69 |  | 0.12 (0.09) | 0.20 |  | | 0.03 (0.06) | | 0.55 |  | -0.07 (0.08) | 0.35 |  | 0.12 (0.08) | 0.14 |
| Chronic job discrimination** | 0 | ref | | |  | | ref | |  | ref | | |  | | ref | |  | ref | |  | ref | |
|  | 1 | -0.02 (0.04) | 0.67 |  | | 0.03 (0.05) | | 0.51 |  | -0.04 (0.07) | 0.63 |  | | -0.05 (0.04) | | 0.23 |  | -0.05 (0.05) | 0.37 |  | -0.00 (0.06) | 0.95 |
|  | 2 | -0.03 (0.04) | 0.49 |  | | -0.03 (0.05) | | 0.49 |  | 0.06 (0.07) | 0.37 |  | | 0.004 (0.04) | | 0.92 |  | 0.04 (0.06) | 0.46 |  | 0.04 (0.06) | 0.54 |
| **Society Level Stress** | | | | | | | | | | | | | | | | | | | | | | |
| Lifetime discrimination |  | 0.002 (0.01) | 0.86 |  | | -0.003 (0.02) | | 0.86 |  | 0.003 (0.02) | 0.89 |  | | -0.01 (0.01) | | 0.47 |  | 0.01 (0.01) | 0.57 |  | -0.01 (0.01) | 0.52 |
| Daily discrimination |  | 0.01 (0.004) | 0.01 |  | | 0.002 (0.005) | | 0.67 |  | 0.02 (0.005) | 0.0007 |  | | 0.001 (0.004) | | 0.77 |  | 0.01 (0.005) | 0.07 |  | -0.001 (0.01) | 0.90 |
| *All models control for age at MIDUS 2, baseline cognition score, race/ethnicity (White, Hispanic, other), education (some college or more vs high school of less), living with partner, income (per 100% above FPL), unemployed (vs employed), retired (vs employed), physical health (self-reported), vigorous physical activity, depression (continuous scale), anxiety (continuous scale) | | | | | | | | | | | | | | | | | | | | | | |
| **Note on chronic job discrimination: higher score represents less frequent reported discrimination (see variable questionnaire/construct) | | | | | | | | | | | | | | | | | | | | | | |

| **Supplemental Table 10. Stress/strain variables and predicting episodic memory and executive function at MIDUS 2 (cross-sectional)** | | | | | | | | | | | | |
| --- | --- | --- | --- | --- | --- | --- | --- | --- | --- | --- | --- | --- |
| Outcome: MIDUS2 Cognition. Higher score represents better baseline cognitive score, lower score represents worse baseline cognitive score | | | | | | | | | | | | |
|  |  | **Episodic Memory** | | | | |  | **Executive Function** | | | | |
| **Scales** | **Tertile** | **Men (n=785-984)** | |  | **Women (n=818-1234)** | |  | **Men (n=785-984)** | |  | **Women (n=818-1234)** | |
|  |  | β (SE) | P-value |  | β (SE) | P-value |  | β (SE) | P-value |  | β (SE) | P-value |
| **Family & Marital Level Stress** | | | | | | | | | | | | |
| Perceived Inequality of family | 0 | ref | |  | ref | |  | ref | |  | ref | |
|  | 1 | -0.11 (0.06) | 0.09 |  | 0.02 (0.07) | 0.71 |  | -0.004 (0.05) | 0.93 |  | 0.005 (0.04) | 0.90 |
|  | 2 | -0.30 (0.10) | 0.003 |  | 0.04 (0.09) | 0.65 |  | 0.02 (0.07) | 0.74 |  | -0.08 (0.05) | 0.14 |
| Marital risk | 0 | ref | |  | ref | |  | ref | |  | ref | |
|  | 1 | -0.05 (0.08) | 0.55 |  | 0.13 (0.09) | 0.15 |  | -0.10 (0.05) | 0.08 |  | 0.04 (0.05) | 0.50 |
|  | 2 | 0.02 (0.07) | 0.76 |  | 0.05 (0.07) | 0.48 |  | -0.12 (0.05) | 0.01 |  | 0.05 (0.04) | 0.24 |
| Family strain | 0 | ref | |  | ref | |  | ref | |  | ref | |
|  | 1 | 0.16 (0.10) | 0.12 |  | 0.12 (0.12) | 0.33 |  | 0.04 (0.07) | 0.60 |  | 0.06 (0.07) | 0.37 |
|  | 2 | 0.09 (0.11) | 0.40 |  | 0.06 (0.12) | 0.60 |  | -0.00 (0.08) | 1.00 |  | -0.001 (0.07) | 0.99 |
| Spouse/ partner strain | 0 | ref | |  | ref | |  | ref | |  | ref | |
|  | 1 | 0.02 (0.23) | 0.95 |  | -0.08 (0.16) | 0.61 |  | 0.09 (0.17) | 0.61 |  | 0.02 (0.09) | 0.84 |
|  | 2 | -0.01 (0.23) | 0.96 |  | -0.01 (0.16) | 0.93 |  | 0.07 (0.17) | 0.67 |  | -0.01 (0.09) | 0.88 |
| **Work Level Stress** | | | | | | | | | | | |  |
| Perceived Inequality at work | 0 | ref | |  | ref | |  | ref | |  | ref | |
|  | 1 | -0.07 (0.07) | 0.29 |  | -0.06 (0.07) | 0.39 |  | -0.12 (0.05) | 0.01 |  | -0.10 (0.05) | 0.03 |
|  | 2 | -0.23 (0.09) | 0.01 |  | -0.04 (0.10) | 0.67 |  | -0.23 (0.07) | 0.0006 |  | -0.15 (0.06) | 0.02 |
| Chronic job discrimination | 0 | ref | |  | ref | |  | ref | |  | ref | |
|  | 1 | 0.05 (0.07) | 0.51 |  | -0.01 (0.07) | 0.93 |  | 0.002 (0.05) | 0.98 |  | -0.04 (0.04) | 0.36 |
|  | 2 | 0.03 (0.07) | 0.70 |  | 0.01 (0.08) | 0.92 |  | -0.02 (0.05) | 0.66 |  | -0.10 (0.05) | 0.03 |
| **Society Level Stress** | | | | | | | | | | | | |
| Lifetime discrimination | cont' | -0.03 (0.02) | 0.18 |  | 0.02 (0.02) | 0.40 |  | -0.04 (0.01) | 0.007 |  | 0.01 (0.01) | 0.17 |
| Daily discrimination | cont' | -0.01 (0.01) | 0.02 |  | -0.005 (0.01) | 0.48 |  | -0.02 (0.002) | <.0001 |  | -0.01 (0.004) | 0.002 |
| *All models control for age at MIDUS 2, baseline cognition score, race/ethnicity (White, Hispanic, other), education (some college or more vs high school of less), living with partner, income (per 100% above FPL), unemployed (vs employed), retired (vs employed), physical health (self-reported), vigorous physical activity, depression (continuous scale), anxiety (continuous scale) | | | | | | | | | | | | |
